# Strain differences in arsenic-induced oxidative lesion via arsenic biomethylation between C57BL/6J and 129X1/SvJ mice

**DOI:** 10.1038/srep44424

**Published:** 2017-03-17

**Authors:** Ruirui Wu, Xiafang Wu, Huihui Wang, Xin Fang, Yongfang Li, Lanyue Gao, Guifan Sun, Jingbo Pi, Yuanyuan Xu

**Affiliations:** 1 Program of Environmental Toxicology, School of Public Health, China Medical University, Shenyang, Liaoning, China; 2 Environment and Non-communicable Disease Research Center, School of Public Health, China Medical University, Shenyang, Liaoning, P. R. China; 3 Experimental Teaching Center, School of Public Health, China Medical University, Shenyang, Liaoning, P. R. China

## Abstract

Arsenic is a common environmental and occupational toxicant with dramatic species differences in its susceptibility and metabolism. Mouse strain variability may provide a better understanding of the arsenic pathological profile but is largely unknown. Here we investigated oxidative lesion induced by acute arsenic exposure in the two frequently used mouse strains C57BL/6J and 129X1/SvJ in classical gene targeting technique. A dose of 5 mg/kg body weight arsenic led to a significant alteration of blood glutathione towards oxidized redox potential and increased hepatic malondialdehyde content in C57BL/6J mice, but not in 129X1/SvJ mice. Hepatic antioxidant enzymes were induced by arsenic in transcription in both strains and many were higher in C57BL/6J than 129X1/SvJ mice. Arsenic profiles in the liver, blood and urine and transcription of genes encoding enzymes involved in arsenic biomethylation all indicate a higher arsenic methylation capacity, which contributes to a faster hepatic arsenic excretion, in 129X1/SvJ mice than C57BL/6J mice. Taken together, C57BL/6J mice are more susceptible to oxidative hepatic injury compared with 129X1/SvJ mice after acute arsenic exposure, which is closely associated with arsenic methylation pattern of the two strains.

Arsenic is one of the most important pollutants ubiquitously existing in the natural and disposed by human activity^1^. This metalloid can be found in water, soil and air in both inorganic forms and organic forms. Drinking water with contamination of arsenic, primarily in the form of inorganic, is considered as a major public health problem. Chronic arsenic exposure due to arsenic-contaminating ground water has been found in many countries and areas^2^. As a human carcinogen, chronic exposure to arsenic has been linked to increased risk of cancers of the skin, lung, bladder, liver and kidney^2^. In addition, associations of chronic arsenic exposure with cardiovascular dysfunction, diabetes, respiratory symptoms, neurological defects, and reproductive issues have been reported^2^,^3^. Another reason for the importance of arsenic in the toxicology field in that the occurrence of acute arsenic poisonings in homicide, suicide or accident^4^,^5^. Acute arsenic poisonings are often linked to ingestion of inorganic arsenic (iAs) and can cause multi-organ (such as the liver) injury and even death^4^,^5^.

Animal model studies, in which exposure conditions can be manipulated and genetic and environmental factors can be controlled, provide valuable information on arsenic health effects and underlying mechanisms. Major developments in genetically modified (i.e. knock-out, knock-in and transgenic) mice over the past few decades have provided a powerful new tool for molecular toxicological and pharmacological research. A classical gene targeting technique is performed in embryonic stem cells from a 129 substrain^6^. Then the 129 chimeras are often mated with C57BL/6 (B6) mice. There are distinct interpretative difficulties as a consequence of the mixed genetic background of mutant mice^7^,^8^,^9^. The offspring of such matings have one set of chromosomes from strain 129 and another from strain B6. Even the mutant mice and their wild-type littermates are not only different at the locus of the target gene but also at other loci^7^. Interpretation of phenotypical alterations based on only the modification of the target gene might lead to an overlook of the effects of other genes, resulting in misinterpretation of results. Therefore, it is important to conduct a background analysis of the inbred mouse strains before the study on selected mutations. There are many reports on the differences between the two commonly used mouse strains (B6 and 129) in gene targeting technique in response to environmental factors and chemicals, such as behavior in an enriched environment^10^, browning propensity of white adipocytes^11^, growth deficit in embryo^12^ and ataxia after ethanol exposure^13^, and susceptibility to anaplasma phagocytophilum^14^.

After a literature review, we found that there is no report on comparison between B6 and 129 mice in response to arsenic. The exact cellular and molecular mechanisms underlying arsenic toxicity are largely unrevealed. However, arsenic is well known to cause the generation of reactive oxygen species (ROS), leading to significant oxidative stress^15^,^16^. Previously, we have observed that B6 mouse strain and a substrain of 129 mice (129X1/SvJ, named as 129 afterwards) are markedly different in blood ratio of reduced glutathione to oxidized glutathione (GSH/GSSG)^17^, a frequently used marker to estimate redox state in the body. Therefore, we assume that the susceptibility to arsenic toxicity and antioxidative response in the two mouse strains are different. Another factor determining arsenic-associated toxicity is arsenic metabolism^18^,^19^,^20^,^21^. Inorganic arsenic undergoes biomethylation in the body, giving rise to methylated metabolites^22^,^23^. These metabolites may be easier to excrete^24^ and show diverse toxic effects^22^,^25^. Arsenic methylation capacity is species dependent, however, the intra-mouse-strain difference is not clear. Thus we also detected arsenic metabolism in B6 and 129 mice. In the present study, we find that alteration of redox potential and hepatic oxidative injury induced by acute arsenic exposure vary in the two mouse strains, which cannot be fully explained by antioxidative response of the two mouse strains, but is closely related to their distinct arsenic methylation capacity.

## Results

### Clinical symptoms and blood glutathione status

All animals survived a single oral dose (5 mg/kg body weight arsenic, 24 h) of sodium arsenite. No obvious clinical symptoms were observed except that B6 mice were less active compared with 129 mice.

**Figure 1:**
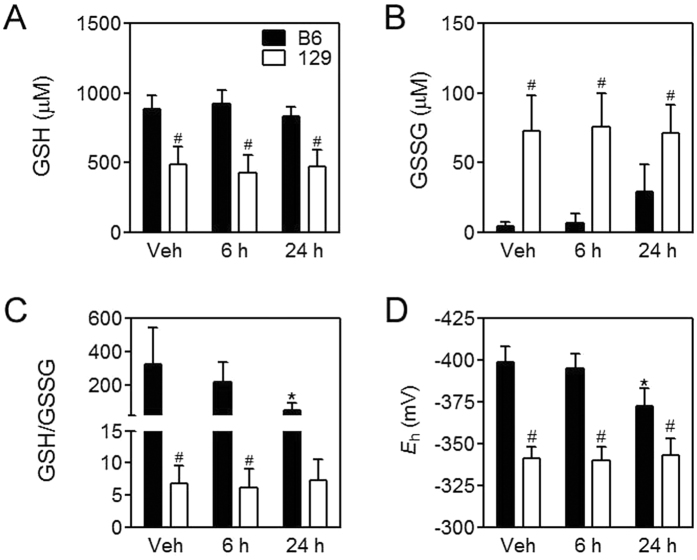
Blood glutathione status. Levels of GSH (**A**) and GSSG (**B**), ratios of GSH to GSSG (GSH/GSSG) (**C**) and GSH *E*_h_ (**D**) in the blood in B6 (closed bars) and 129 (open bars) mice at the indicated intervals after arsenic challenge. Values are expressed as mean + SD. *p <* *0.05, compared with vehicle (Veh) of the same strain; ^#^*p *<* *0.05, compared with time-matched B6 mice. n = 5–6.

Blood GSH/GSSG ratio is the most important and frequently used index to estimate redox state in the body^26^,^27^. As arsenic is a well-known oxidative stressor, we first examined blood glutathione status in the two mouse strains. 129 mice showed lower blood GSH levels, higher GSSG levels, and thus significantly lower GSH/GSSG ratios under basal conditions compared with B6 mice (Fig. 1A–C). After arsenic challenge, B6 mice showed a significant decrease in blood GSH/GSSG due to the elevation of GSSG levels at 24 h, while 129 mice did not showed any significant change of blood glutathione status (Fig. 1A–C). Blood GSH redox potential (*E*_h_) values (Fig. 1D) also indicate decreased reducing capacity in B6 mice but not in 129 mice after arsenic challenge at 24 h.

### Hepatic injury and expression of antioxidant enzymes

**Figure 2:**
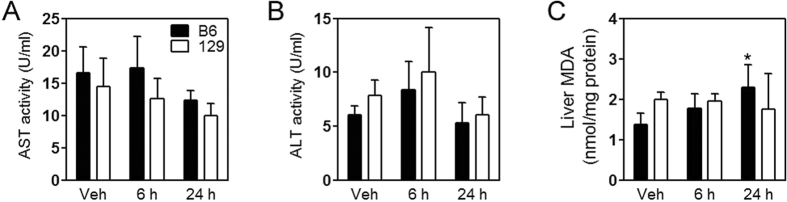
Analysis of acute hepatic injury induced by arsenite in mice. Levels of AST (**A**) and ALT (**B**) in the plasma and MDA content (**C**) in the liver in B6 (closed bars) and 129 (open bars) mice at the indicated intervals after arsenic challenge. Values are expressed as mean + SD. *p <* *0.05, compared with vehicle (Veh) of the same strain. n = 5–6.

**Figure 3:**
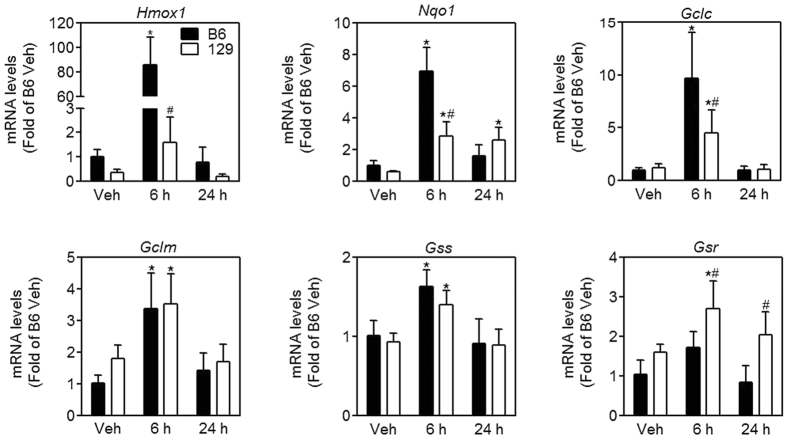
Expression of genes encoding antioxidative enzymes in the liver. mRNA levels of *Homx1, Nqo1, Gclc, Gclm, Gss* and *Gr* in the liver in B6 (closed bars) and 129 (open bars) mice at the indicated intervals after arsenic challenge. Values are expressed as mean + SD. *p <* *0.05, compared with vehicle (Veh) of the same strain; ^#^*p *<* *0.05, compared with time-matched B6 mice. n = 5–6.

The liver is one of the primary targets for acute arsenic toxicity *in vivo*^4^,^5^,^28^. We examined plasma markers for liver injury, aspartate aminotransferase (AST) and alanine aminotransferase (ALT). There is no significant alteration in plasma levels of AST (Fig. 2A) or ALT (Fig. 2B). However, the content of malondialdehyde (MDA) in the liver was significantly elevated at 24 h after oral arsenite administration in B6 mice but remained unchanged in 129 mice (Fig. 2C), which indicates an oxidative liver injury in B6 mice but not in 129 mice. Furthermore, we tested expression of antioxidant enzymes in the liver (Fig. 3). mRNA levels of all those genes (*Hmox1* encoding heme oxygenase 1, *Nqo1* encoding NAD(P)H dehydrogenase, quinone 1, *Gclc* encoding glutamate-cysteine ligase catalytic subunit, *Gclm* encoding glutamate-cysteine ligase modifier subunit, *Gss* encoding glutathione synthetase, and *Gsr* encoding glutathione reductase) were induced by arsenic in both mice strains, and reached the maximum at 6 h. 129 mice showed lower transcription of *Homx1, Nqo1* and *Gclc*, but higher transcription of *Gsr* compared with B6 mice (Fig. 3).

### Hepatic arsenic metabolism

**Figure 4:**
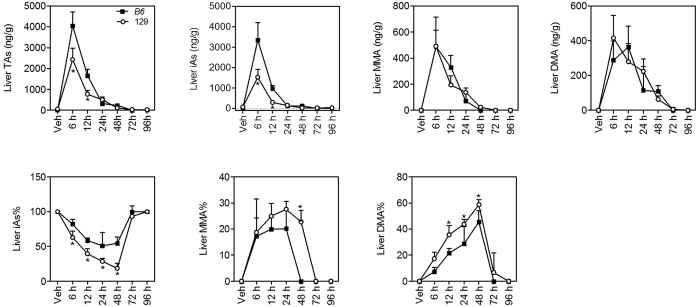
Arsenic concentrations and profiles in the liver in B6 (closed squares) and 129 (open circles) mice. TAs was calculated as the sum of iAs, MMA and DMA. Values are expressed as mean + SD. *p <* *0.05, compared with time-matched B6 mice. n = 5–6.

Metabolism of inorganic arsenic is one of the key factors determining arsenic-associated health effects^18^,^19^,^20^,^21^. The liver plays a primary role in arsenic metabolism, including arsenic methylation and arsenic export. Hepatic levels of different arsenicals [iAs, methylarsonic acid (MMA) and dimethylarsinic acid (DMA)] and total arsenic (TAs) were elevated 6 h after arsenic administration and went back towards basal levels after 48 h in both mouse strains (Fig. 4). Of note, hepatic levels of iAs and TAs in 129 mice were lower than B6 mice at 6 h and 12 h, and close to B6 mice at 24 h after arsenic challenge (Fig. 4). 129 mice also showed significantly lower iAs% (from 6 h to 48 h) but higher DMA% (from 12 h to 48 h) in the liver compared with B6 mice (Fig. 4). MMA% in the liver was not significantly different between the two mouse strains except at 48 h, when hepatic MMA was more than 20% of total arsenic in 129 mice but below limit of detection in B6 mice (Fig. 4). These data suggest a stronger arsenic methylation capacity in the liver of 129 mice.

**Figure 5:**
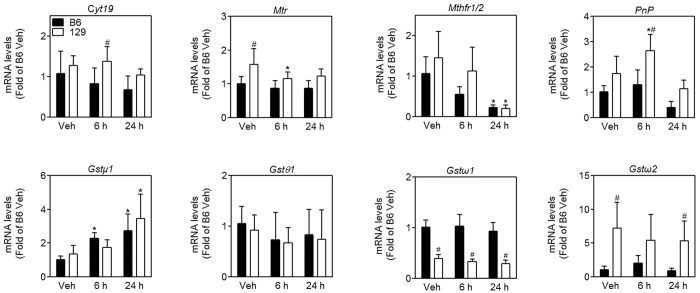
Expression of genes encoding enzymes involved in arsenic methylation in the liver in B6 (closed bars) and 129 (open bars) mice. mRNA levels genes encoding enzymes involved in arsenic methylation (*Cyt19, Mtr* and *Mthfr1*/*2*) and reduction (*Pnp, Gstμ1, Gstθ1, Gstω1* and *Gstω2*). Values are expressed as mean + SD. *p <* *0.05, compared with vehicle (Veh) of the same strain; ^#^*p *<* *0.05, compared with time-matched B6 mice. n = 5–6.

**Figure 6:**
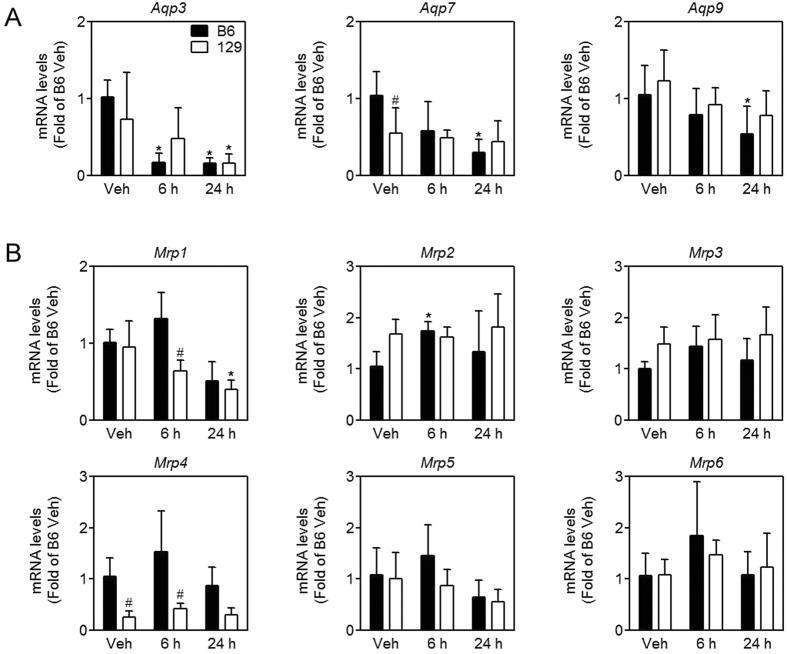
Expression of genes encoding arsenic transporters in the liver in B6 (closed bars) and 129 (open bars) mice. mRNA levels of arsenic influx transporters (**A**), MRP arsenic efflux pumps (**B**). Values are expressed as mean + SD. *p <* *0.05, compared with vehicle (Veh) of the same strain; ^#^*p *<* *0.05, compared with time-matched B6 mice. n = 5–6.

Then transcription of genes encoding enzymes involved in arsenic biomethylation and transport was determined. As expected, mRNA levels of genes involved in arsenic methylation, including arsenic (+3 oxidation state) methyltransferase (*Cyt19*), 5-methyltetrahydrofolate-homocysteine methyltransferase (*Mtr*), purine nucleoside phosphorylase (*Pnp*) and glutathione S-transferase omega 2 (*Gstω2*), were higher in the liver of 129 mice than B6 mice (Fig. 5). mRNA levels of typical arsenic influx transporters, such as *aquaglyceroporin (Aqp) 3, Aqp7* and *Aqp9*, showed a decreasing trend over time after arsenic challenge, but were not significantly different between B6 and 129 mice (Fig. 6A). Among six arsenic efflux transporters that we determined, mRNA levels were significantly lower for *multidrug resistance-associated protein (Mrp) 1* and *Mrp4* in the liver in 129 mice than B6 mice after arsenic challenge (Fig. 6B). According to cycle time of reverse transcription-quantitative polymerase chain reaction (RT-qPCR), mRNA levels for *Mrp2* and *Mrp3* were much more abundant than other *Mrps* in the liver (22 vs 28–30), indicating *Mrp2* and *Mrp3* may play a major role in arsenic efflux in the liver. However, there was no difference in mRNA levels of *Mrp2* and *Mrp3* between the two strains.

### Arsenic metabolites in the blood and urine

**Figure 7:**
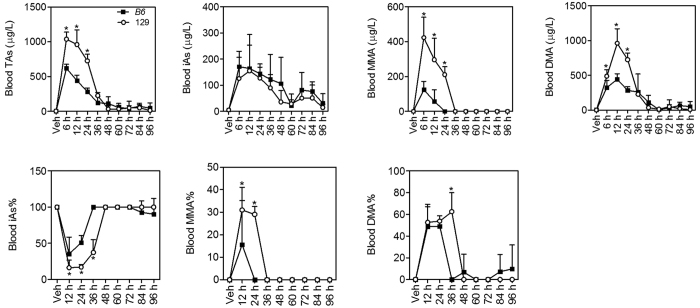
Arsenic concentrations and profiles in the blood in B6 (closed squares) and 129 (open circles) mice. TAs was calculated as the sum of iAs, MMA and DMA. Values are expressed as mean + SD. *p <* *0.05, compared with time-matched B6 mice. n = 5–6.

**Figure 8:**
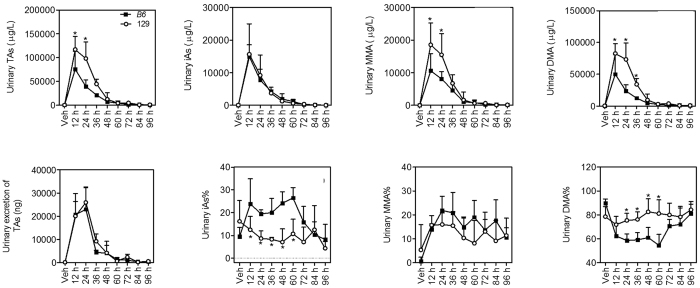
Arsenic concentrations and profiles in the urine in B6 (closed squares) and 129 (open circles) mice. TAs was calculated as the sum of iAs, MMA and DMA. Urinary excretion of TAs was calculated by multiple urinary concentrations of TAs and urine amount. Values are expressed as mean + SD. *p <* *0.05, compared with time-matched B6 mice. n = 5–6.

Arsenic methylation has been found to facilitate excretion of this metalloid^24^. It appears that 129 mice have a higher arsenic methylation capacity and excrete arsenic from the liver more quickly than B6 mice. To further test this idea, arsenic concentrations and profiles in the blood and urine were examined. In both mouse strains, TAs in the blood and urine were significantly elevated in 36 h after arsenic challenge compared with Vehicle (Veh) (Figs 7 and 8). 129 mice showed higher concentrations of different arsenicals and TAs in the blood (Fig. 7) and urine (Fig. 8) than B6 mice in 36 h and 24 h after arsenic challenge, respectively. However, urinary excretion of TAs at each time point was not significantly different between the two mouse strains (Fig. 8) because 129 mice excreted less urine than B6 mice in 48 h (Supplemental Information Figure S1). Arsenic speciation in the blood (Fig. 7) showed lower iAs% but higher MMA% and DMA% from 6 h to 36 h in 129 mice than B6 mice. In the urine, iAs% was significantly lower but DMA% was significantly higher in 129 mice than B6 mice in 60 h after arsenic challenge (Fig. 8). There was no significant difference in urinary MMA% between the two mice strains. All indicate a stronger arsenic methylation capacity in 129 mice.

## Discussion

In the present study, we compared the oxidative lesion due to acute arsenic exposure in the two inbred mouse strains that are commonly used in the traditional gene targeting technique. Overall, B6 mice showed more obvious oxidative stress in the liver. These data indicate that susceptibility to arsenic intoxication is closely related to genetic background of the mouse.

Though different analytical methods used to detect GSH and GSSG give rise to variation in reported values of GSH and GSSG in blood, with proper sample handling and determination, the physiological concentration of GSH in blood is typically at millimolar, whereas that of GSSG at micromolar^29^. According to previous studies, some mouse strains, such as C57BL/6^17^,^30^, BABL/C^31^, DBA/2^30^ and Oncins France 1 (OF1)^32^,^33^, showed concentrations of GSH and GSSG within the above ranges. However, we observed that other mouse strains, such as A/J and 129, showed much higher concentrations of GSSG than usual (approximately 80–200 micromolar) in blood^17^. The exact mechanism underlying this phenomenon is unclear, but may be related to strain-dependent susceptibility to oxidative damage and diseases. Therefore, we assume that B6 mice are different from 129 mice in susceptibility to arsenic toxicity. Here we found that B6 mice exhibited more obvious oxidative stress according to blood glutathione status and liver MDA levels. The blood GSH/GSSG ratio was decreased due to increased GSSG levels in B6 mice but remained stable in 129 mice after arsenic challenge. Higher expression of *Gsr*, which catalyzes reduction of GSSG to GSH in 129 mice after arsenic challenge may contribute to this phenomenon. However, 129 mice showed higher blood GSSG levels with higher expression of *Gsr* than B6 mice, suggesting other factors are involved in maintaining balance of GSH and GSSG. As reported previously, nicotinamide adenine dinucleotide phosphate (NADPH) is also key to reduction of GSSG to GSH as proton donor^34^; glucose-6-phosphate dehydrogenase (G6PD) acts as the rate-limiting enzyme for NADPH generation^35^.

Transcription of genes involved in antioxidative defense was induced by arsenic challenge. These genes are all nuclear factor (erythroid-derived 2)-like 2 (NRF2) dependent, containing the antioxidant response element (ARE) in the promoter region^36^,^37^,^38^,^39^. Generally, NRF2-ARE pathway is considered to mediate cellular defense response. Paradoxically, NRF2-ARE pathway is also activated by toxic chemicals that induce oxidative stress, such as arsenic^40^,^41^. Arsenic activates NRF2-ARE pathway by increasing association between Keap1 and Cul3, resulting in decreased E3 ubiquitin ligase activity and disrupted degradation of NRF2^40^. This is different from the mechanisms of NRF2 activation by beneficial natural-occurring compounds. Under such a scenario, enhanced transcription of the NRF2 downstream genes appears as a reflection of arsenic-induced oxidative lesions. Therefore it is not a surprise to see that hepatic mRNA levels of *Homx1, Nqo1* and *Gclc* were lower in 129 mice compared with B6 counterparts, which is consistent with the internal doses of arsenic in the liver of the two mouse strains.

Most ingested inorganic arsenic is absorbed from the gastrointestinal tract^42^, biomethylated predominantly in the liver^22^, and largely excreted from the urine. The metabolism of arsenic is considered to affect its toxicity^18^,^19^,^20^,^21^. AQP3, AQP7 and AQP9 are transporters for cellular uptake of arsenicals^43^,^44^. AQP9 is the most effective transporter of arsenite among AQP family^45^,^46^. It is expressed predominantly in hepatocytes^44^ and also mediates excretion of arsenic from the liver^46^,^47^. In support of this, *Aqp9*-knockout mice show a decreased efficiency of arsenic clearance and are more susceptible to arsenic toxicity^47^. Consistent with previous reports, AQP9 is the most predominant AQPs in the liver of B6 and 129 mice in the present study. mRNA levels of *Aqp9* are slightly higher in 129 mice, but not significantly different from B6 mice, indicating that difference in hepatic arsenic concentrations between the two mouse strains is not caused by arsenic influx. ATP-binding cassette (ABC) proteins have a central role in active transport of heavy metals. The multidrug resistance protein (MRP) is a member of ABC family with established functions as arsenic efflux pumps^46^. Though MRP1 and MRP2 are responsible for the excretion of arsenic metabolites in the form of arsenic-GSH conjugates, they are not localized to the basolateral membrane of hepatocytes^48^,^49^. Of basolateral MRPs, *Mrp3, Mrp4* and *Mrp6* are reported to be significantly expressed at mRNA levels in the liver of mice^50^. MRP6 might mediate hepatic efflux of arsenic-GSH conjugates and other arsenic metabolites^46^, but was expressed at quite low mRNA levels in the present study. MRP3 was predominantly expressed at mRNA levels in the liver, but might not mediate efflux of inorganic or methylated arsenicals^46^. MRP4 is suggested to play a vital role in elimination of methylated arsenicals^46^,^51^,^52^, but its transcription was not consistent with the results of hepatic arsenic excretion of the two mouse strains. Thus, strain difference in response to arsenic is not likely to be caused by arsenic transporters in the liver.

Two pathways are proposed for arsenic biomethylation^53^. The classical one suggests that arsenate is imported into cells by phosphate transport proteins^53^,^54^, and subjected to successive reduction and oxidative methylation, giving rise to MMA and DMA^22^,^23^. The other theory suggests that after reduction from the pentavalent to the trivalent, arsenicals bind to glutathione^55^ or certain proteins^56^ prior to further methylation. In both theories, CYT19 is the primary enzyme that can catalyze the methylation of arsenite to its methylated forms^57^. This enzyme is able to methylate arsenite using S-Adenosyl-methionine (SAM) as methyl donor and a variety of endogenous reductants^58^,^59^. 5-methyltetrahydrofolate-homocysteine methyltransferase (MTR) is indicated to influence arsenic methylation via one-carbon metabolism, which produces SAM, the methyl donor for arsenic methylation^60^. In addition, epidemiological and *in vitro* studies suggest that others enzymes, such as glutathione S-transferase (GST) omega 1 (GSTω1), GST omega 2 (GSTω2), GST Mu 1 (GSTμ1), GST theta 1 (GSTθ1) and purine nucleoside phosphorylase (PNP) might be involved in reduction of pentavalent arsenicals and thus affect arsenic methylation^61^,^62^,^63^,^64^,^65^. Lower arsenic methylation capacity leads to higher proportions of inorganic arsenic and MMA in the urine, and has been associated with higher retention of arsenic in the body^23^,^24^ and increased susceptibility to arsenic-induced adverse health effects^18^,^19^,^20^,^21^. In the present study, percentages of methylated arsenicals (MMA% and DMA%) were higher in the blood and urine compared with the liver (Fig. 4 vs Figs 7 and 8), supporting the idea that arsenicals that undergo biomethylation are easier to excrete from the body. Arsenical profiles in the liver, blood and urine all indicate a higher arsenic methylation capacity in 129 mice than B6 mice. In line with this, many above-mentioned enzymes participating in arsenic biomethylation are expressed at higher mRNA levels in the liver of 129 mice. High arsenic methylation capacity in 129 mice leads to a quick hepatic arsenic excretion, which may serve as a protective mechanism for the liver of 129 mice in acute arsenic poisoning. On the other hand, arsenic methylation in chronic arsenic exposure is suggested to facilitate tumorigenesis. Trivalent MMA and trivalent DMA produced in the process of arsenic methylation are more toxic and biologically active^25^,^66^,^67^. Consumption of SAM by arsenic methylation can lead to a global DNA hypomehtylation^68^, which has been found in a wide variety of cancers^69^. An obstacle in research of mechanisms for arsenic carcinogenesis is the distinct species susceptibility to arsenic toxicity, which is associated with arsenic methylation to some extent^68^. During chronic arsenic exposure, 70–90% of urinary total arsenic in humans is methylated^70^, whereas in acute arsenic poisoning, urinary methylated arsenicals drop to 45–75%^71^,^72^. Our data indicate that both B6 and 129 mice are more efficient in arsenic methylation than humans in response to acute arsenic exposure. B6 strain is relatively close to humans and may serve as a better mouse model to study arsenic carcinogenesis with respect to arsenic methylation, which requires further study. One limitation of the present study is that enzymes involved in arsenic metabolism were only determined at mRNA levels but not protein or enzyme activity levels, which may be more informative for future studies.

Taken together, the susceptibility to oxidative stress due to acute arsenic toxicity is different between B6 and 129 mice, which can be explained, at least in part, by characteristics of arsenic methylation. The distinct arsenic methylation patterns of the two inbred mouse strains may shed light on choosing a proper mouse model to study carcinogenic effects of chronic arsenic exposure.

## Methods

### Animals and experimental design

Pathogen-free adult male B6 and 129 mice (8- to 12-week old) were obtained from Model Animal Research Center of Nanjing University (Najing, China). Mice were maintained in a regulated environment (22 ± 1 °C) with a 12 h: 12 h light: dark cycle, and were fed standard chow diet (Shukebeita Specific Pathogen Free Mouse Maintenance Diet, Xietong Organism, Jiangsu, China). According to standardization administration of P. R. China (reference number GB 14924.2-2001), the arsenic is less than 0.7 μg/g dry food. Experiments were carried out under protocols approved by China Medical University (Authorization Reference No. 14008 M). All methods were performed in accordance with the relevant guidelines and regulations. Sodium arsenite (NaAsO_2_) was purchased from Sigma Aldrich (S7400, St. Louis, USA).

Each strain of mice was randomly divided into two groups. After overnight fasting, arsenic-treated mice were gavaged with 5 mg/kg body weight of arsenic (equivalent to 8.67 mg/kg body weight of NaAsO_2_) next morning, which did not cause death in both mouse strains according to the preliminary study. Control mice were gavaged with equivalent volume of normal saline solution. Animals were closely monitored for clinical symptoms after gavaged, and sacrificed by carbon dioxide anesthesia for sample collection. Immediately after anesthesia, blood was collected from the orbital sinus, and mixed with heparin. Dissection of mice was conducted on ice after perfusion via the heart with ice-cold normal saline solution. Tissues were ligated, rinsed with ice-cold normal saline solution, snap-frozen in liquid nitrogen and stored at −80 °C until use. For analysis of arsenic metabolism, animals were kept individually in metabolic cages (SN-783, Ishizawa Corporation of Medical Implement, Tokyo, Japan) with free access to food and water. Urine and tail-vein blood samples were collected at indicated time points and stored at −80 °C.

### Blood GSH and GSSG

Levels of blood GSH and GSSG were measured with BIOXYTECG GSH/GSSG-412 kit (OxisResearch, Portland, USA) according to manufacturer’s instruction. Heparin blood was immediately mixed with or without 1-methel-2-vinyl-pyridim trifluoromethane sulfonate (M2VP) for measurement of GSSG and total glutathione (GSH + GSSG), respectively. Levels of GSH = total glutathione − (2 × GSSG).

### Calculation of *E*_h_

*E*_h_ values, expressed as mV, were calculated from the Nernst Equation: *E*_h_ = *E*_o_ + *RT*/*nF* ln ([acceptor]/[donor]^2^)^26^. For blood GSH/GSSG pairs, the equation was simplified to *E*_h_ = −240 − (59.1/2) lg([GSH]^2^/[GSSG]), assuming that pH was 7.0. A less negative value indicates a more oxidized redox state.

### Plasma biochemistry

The plasma was obtained after centrifugation (2000 g, 4 °C, 10 min) of heparin-containing blood samples. Biochemical parameters in the plasma were measured using kits from Nanjing Jiancheng Bioengineering Institute (Nanjing, China) according to the manufacturer’s instruction within a week after sample collection. For AST and ALT determination, the plasma was incubated with corresponding substrate at 37 °C for 30 min before further incubation with a 2,4-dinitrophenylhydrazine at 37 °C for 20 min. The reaction was stopped by adding 0.4-N NaOH and absorbance was read at a wavelength of 515 nm. The ALT and AST levels were determined using pyruvate as a standard.

### Hepatic MDA

MDA levels were measured by the thiobarbituric acid-reactive substances assay. The liver sample was homogenized by ice-cold beads with Tissuelyzer II (Qiagen, USA) in 9 volumes of pH 7.4 cold Tris-HCl buffer. The homogenate was centrifuged at 4 °C, 10,000 g for 15 min. Then the supernatant was used for the determination of MDA according to the manufacturer’s instructions (Nanjing Jiancheng Bioengineering Institute). Protein concentrations determined with BCA Protein Assay Kit (Beyotime, Shanghai, China) were used for adjustment of hepatic MDA levels.

### RT-qPCR

The mRNA levels of genes of interest were determined using RT-qPCR as previously described^17^. Total RNA was isolated using TRIzol reagent (Invitrogen, Carlsbad, USA) and reversely transcribed to cDNA using Prime Script RT reagent Kit (Takara, Dalian, China). SYBR Premix EX Taq Kit (Takara) was used to assess cDNA amplifications. Real-time fluorescence was detected using an QuantStudio 6 Flex real-time PCR system (Applied Biosystems). Data were analyzed using the delta-delta cycle time method. Primers were designed with Primer BLAST online (http://www.ncbi.nlm.nih.gov/tools/primer-blast) and obtained from Sigma-Aldrich. Information on gene-specific primers is provided in the Supplementary Information Table S1.

### Arsenic determination

Levels of arsenic species, including iAs, MMA, DMA and trimethylarsine oxide (TMAO), in the liver, blood and urine were determined with cold trap-hydride generation-atomic absorption spectrophotometer (CT-HG-AAS) (ASA-2SP-AA 6800, Shimadzu, Kyoto, Japan) system as previously reported^73^,^74^. For arsenic speciation, all samples were digested with NaOH (urine, 2-N NaOH; blood and liver, 4-N NaOH) solutions at 100 °C for 3 h. TMAO was undetectable or below limit of detection in all samples. Levels of TAs were calculated as the sum of arsenic species. The reliability of arsenic determination was checked by the analytical recoveries of added arsenic species. Spiking control of liver, blood and urine samples with the known amounts of iAs, MMA, DMA, and TMAO (10 μg/L, respectively) were assessed, and the recoveries of iAs, MMA, DMA, and TMAO were ranged from 83% to 112%. Solutions of MMA (GBW08668, 0.335 μmol/g) and DMA (GBW08669, 0.706 μmol/g) were purchased from Beijing Zhongbiao Technology. TMAO (sc-475468) was purchased from Santa Cruz Biotechnology (Santa Cruz, USA). Quality control of arsenic speciation included the analysis of standard reference material of freeze-dried urine (GBW09115, National Institute of Metrology, Beijing, China). The certificated amounts for iAs, MMA and DMA were 0.758 nmol, 1.03 nmol and 4.3 nmol, respectively. The values measured in our laboratory were (0.731 ± 0.084) nmol, (0.963 ± 0.104) nmol and (4.01 ± 0.38) nmol, respectively.

### Statistical Analysis

All statistical analyses were performed using Graphpad Prism 5 (Graphpad Software Inc., San Diego, CA), with *p *<* *0.05 considered significant. Statistical differences were determined by two-way analysis of variance (ANOVA), followed by Dunnett’s post-hoc comparison tests. All data are expressed as mean with standard deviation (SD). For statistical analysis of arsenic metabolites, limit of detection (LOD) was used to replace the value below LOD.

## Additional Information

**How to cite this article:** Wu, R. *et al*. Strain differences in arsenic-induced oxidative lesion via arsenic biomethylation between C57BL/6J and 129X1/SvJ mice. *Sci. Rep.* **7**, 44424; doi: 10.1038/srep44424 (2017).

**Publisher's note:** Springer Nature remains neutral with regard to jurisdictional claims in published maps and institutional affiliations.

## Supplementary Material

Supplementary Information Table S1 and Figure S1
