# Supplementary material for: Strain differences in arsenic-induced oxidative lesion via arsenic biomethylation between C57BL/6J and 129X1/SvJ mice
Source: Sci Rep. 2017 Mar 17;7:44424. doi: 10.1038/srep44424 (PMC5355880; doi:10.1038/srep44424)
Supplement: Supplementary Information Table S1 and Figure S1 [file srep44424-s1.pdf]

Supplementary Information

Strain differences in arsenic-induced oxidative lesion via arsenic biomethylation between C57BL/6 and 129X1/SvJ mice

Ruirui Wu, Xiafang Wu, Huihui Wang, Xin Fang, Yongfang Li, Lanyue Gao, Guifan Sun, Jingbo Pi*, Yuanyuan Xu*

Email: yyxu@ cmu.edu.cn; jbpi@cmu.edu.cn

Table S1. Genes and primers for real time RT-qPCR

Gene	GenBank Accession No.	Primers (5’→3’)
Homx1	NM_010442.2	Forward: CCTCACTGGCAGGAAATCATC
		Reverse: CCTCGTGGAGACGCTTTACATA
Nqo1	NM_008706.5	Forward: TATCCTTCCGAGTCATCTCTAGCA
		Reverse: TCTGCAGCTTCCAGCTTCTTG
Gclc	NM_010295.2	Forward: TGGCCACTATCTGCCCAATT
		Reverse: GTCTGACACGTAGCCTCGGTAA
Gclm	NM_008129	Forward: ACATTGAAGCCCAGGATTGG
		Reverse: CCCCTGCTCTTCACGATGAC
Gs	NM_008180.2	Forward: TGCGGTGGTGCTACTGATTG
		Reverse: CGGCACGCTGGTCAAATAT
Gr	NM_010344.4	Forward: GCACTTGCGTGAATGTTGGA
		Reverse: CGTGATCGTGCATGAATTCC
Cyt19	NM_020577.2	Forward: GGACGTGGAGATCGTGAGTC

		Reverse: CTGCAGACTTTCCCGGATGT
Mtr	NM_001081128.3	Forward: AACACTTGGCCTACCGGATG
		Reverse: TCTGGCCTTTCCACAGATGG
Mthfr1/2	NM_001161798.1;	Forward: ACCTGAAAAGCAAGTCCCCC
	NM_010840.3	Reverse: GGCAGGTTACTCTGTAGCCA
Pnp	NM_013632.4	Forward: AAGTTCTGGATGCCGGGAAA
		Reverse: AGAGAGCTACTTGGACCCCA
Gstμ1	NM_010358.5	Forward: CAAGATCACCCAGAGCAATGC
		Reverse: CATCCAGGTGGTGCTTTCG
Gstθ1	NM_008185.3	Forward: CTGTACCTGGATCTGCTGTCG
		Reverse: TAGCCACACTCTCACACAGGG
Gstω1	NM_010362.3	Forward: GGATGAGGCCTACCCAGAGA
		Reverse: CAAAGGCGGCACCTTAGAGA
Gstω2	NM_026619.2;	Forward: GTTCCAGGGGAGTAGTTGCC
	NM_030051.1	Reverse: ACAGTCGACAGCACTCTTGC
β-actin	NM_007393.5	Forward: CACTGTCGAGTCGCGTCC
		Reverse: TCATCCATGGCGAACTGGTG
Gapdh	NM_001289726.1;	Forward: GTATGACTCCACTCACGGCAAA
	NM_008084.3	Reverse: GGTCTCGCTCCTGGAAGATG
Aqp3	NM_016689.2	Forward: GCTTTTGGCTTCGCTGTCAC
		Reverse: TAGATGGGCAGCTTGATCCAG
Aqp7	NM_007473.4	Forward: AATATGGTGCGAGAGTTTCTGG

		Reverse: AACCCAAGTTGACACCGAGAT
Aqp9	NM_022026.3;	Forward: TGGTGTCTACCATGTTCCTCC
	NM_001271843.1	Reverse: AACCAGAGTTGAGTCCGAGAG
Mrp1	NM_008576.3	Forward: CATGTGGACGTGTTTCGAGATT
		Reverse: CACGAGCTGAACAAGCACAAG
Mrp2	NM_013806.2	Forward: CGACCATCCGGAACGAGTT
		Reverse: CCGCTGTCTAGGACCATTATCT
Mrp3	NM_029600.3	Forward: AGTTTGGCCCGAGCTGTTTA
		Reverse: CAGCCGACAGTGGGTCATC
Mrp4	NM_001163676.1	Forward: ACGCTTCCCAGACTTTGCA
		Reverse: AACAACACGGGAGCCTTCAG
Mrp5	NM_013790.2;	Forward: CCCTTCCGGACCACTACCA
	NM_176839.1	Reverse: TGTAGGAGAAAAGTCCAGCATTGT
Mrp6	NM_021050.2	Forward: CAGCTCAAACAACTGGAATCTGA
		Reverse: GCTCGAAGTGTCCAGAGTCCTT

All primers are from Sigma-Aldrich.

Figure

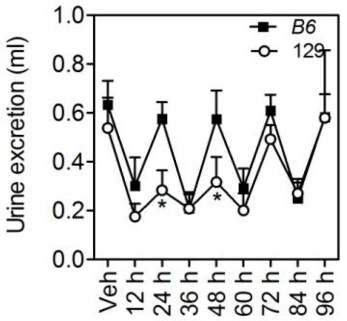

Figure S1 Urine excretion by B6 (closed squares) and 129 (open circles) mice.
